# Texture analysis- and support vector machine-assisted diffusional kurtosis imaging may allow *in vivo* gliomas grading and IDH-mutation status prediction: a preliminary study

**DOI:** 10.1038/s41598-018-24438-4

**Published:** 2018-04-17

**Authors:** Sotirios Bisdas, Haocheng Shen, Steffi Thust, Vasileios Katsaros, George Stranjalis, Christos Boskos, Sebastian Brandner, Jianguo Zhang

**Affiliations:** 1 Department of Neuroradiology, The National Hospital for Neurology and Neurosurgery, University College London NHS Foundation Trust, London, UK; 2 Department of Brain Repair and Rehabilitation, Institute of Neurology, University College London, London, UK; 3 Institute of Healthcare Engineering, University College London, London, UK; 4 Computing, School of Science and Engineering, University of Dundee, Dundee, UK; 5 Department of Neuroradiology, General Anti-Cancer and Oncological Hospital of Athens “St.Savvas”, Athens, Greece; 6 Department of Neurosurgery, , University of Athens, Evangelismos Hospital, Athens, Greece; 7 Department of Radiation Oncology, Athens Medical Center, Athens, Greece; 8 Division of Neuropathology, The National Hospital for Neurology and Neurosurgery, University College London NHS Foundation Trust, London, UK; 9 Department of Neurodegenerative Disease, Institute of Neurology, University College London, London, UK

## Abstract

We sought to investigate, whether texture analysis of diffusional kurtosis imaging (DKI) enhanced by support vector machine (SVM) analysis may provide biomarkers for gliomas staging and detection of the IDH mutation. First-order statistics and texture feature extraction were performed in 37 patients on both conventional (FLAIR) and mean diffusional kurtosis (MDK) images and recursive feature elimination (RFE) methodology based on SVM was employed to select the most discriminative diagnostic biomarkers. The first-order statistics demonstrated significantly lower MDK values in the IDH-mutant tumors. This resulted in 81.1% accuracy (sensitivity = 0.96, specificity = 0.45, AUC 0.59) for IDH mutation diagnosis. There were non-significant differences in average MDK and skewness among the different tumour grades. When texture analysis and SVM were utilized, the grading accuracy achieved by DKI biomarkers was 78.1% (sensitivity 0.77, specificity 0.79, AUC 0.79); the prediction accuracy for IDH mutation reached 83.8% (sensitivity 0.96, specificity 0.55, AUC 0.87). For the IDH mutation task, DKI outperformed significantly the FLAIR imaging. When using selected biomarkers after RFE, the prediction accuracy achieved 83.8% (sensitivity 0.92, specificity 0.64, AUC 0.88). These findings demonstrate the superiority of DKI enhanced by texture analysis and SVM, compared to conventional imaging, for gliomas staging and prediction of IDH mutational status.

## Introduction

Exciting advances and an improved understanding of the brain has been facilitated by diffusion-weighted (DWI) MRI, which for brain tumors supplies a measure of tumor cellularity based on the restriction of the free diffusion of water in proliferating tissue^1^. The tumor cellularity, as estimated through diffusion restriction, has been correlated with the degree of tumor malignancy^2^. Noteworthy, the clinically disseminated DWI is based on the assumption of a Gaussian distribution of signal values interpreted as uniform diffusion of the water molecules in a certain direction, which ideally would be the case only in a bucket of water. However *in vivo*, the water molecules in brain diffuse through a highly heterogeneous environment leading to deviation from the assumed Gaussian distribution. Diffusion kurtosis imaging (DKI) is an attempt to account for this variation and in a more refined approach overcomes this problem by quantifying the deviation from the Gaussian distribution of diffusion properties in brain tissue^3^.

Raab *et al*. demonstrated differences for mean kurtosis (MK) and ADC values in WHO grade 2–4 astrocytomas, with statistically significant higher MK values for high-grade gliomas (HGGs)^4^. van Cauter *et al*. showed a high accuracy for the distinction of HGGs from the low-grade gliomas (LGGs) based on mean axial and radial kurtosis values^5,6^. Mean kurtosis has outperformed diagnostically the remainder of the diffusion metrics for grading (WHO grade 2–4) and predicting Ki-67 as a measure of cellularity^7^. Tietze *et al*. proposed the usage of MK′ (derived from a fast kurtosis sequence) for glioma grading and demonstrated that the mean value of MK′ in the tumor core was significantly higher in HGGs than in LGGs^8^. Common outcome in the aforementioned studies is the degree of the diagnostic accuracy of DKI for the conventional glioma grading (HGG vs. LGG). In light of the 2016 update of the WHO brain tumor classification that stipulates an integrated, ‘layered’ diagnosis based on histological and molecular features^9^, isocitrate dehydrogenase 1 and 2 (IDH1 and IDH2) mutations play a key role in the classification of gliomas. Elkhaled at al. found a significantly negative relationship between IDH-mutation status, as identified via 2-HG (2-hydroxyglutatarate) levels in tissue, and the rather lump apparent diffusion coefficient – ADC)^10^. Xiong *et al*. demonstrated significantly lower minimum ADC in IDH wild-type oligodendrogliomas than in IDH-mutant by using DTI^11^. According to the new biomarker-driven WHO classification, a proportion of these tumours, in particular those without IDH mutation and 1p/19q co-deletion, probably have represented IDH wild-type glioblastomas. Hence, the results of this study contradict the previous work by Elkhaled *et al*.^10^ but essentially highlight that (i) the methodology for obtaining DWI parameters in tumor plays a crucial role given the spatial heterogeneity and (ii) the ADC might be not the most appropriate parameter to gauge any altered diffusion properties related to the IDH mutation, and DKI should be employed to improve the diagnostic accuracy^12^. Preliminary evidence supports the potential role for DKI as a biomarker in the context of the new integrated glioma diagnosis^13^.

Apart from the DWI studies, Patel *et al*.^14^ made an important contribution by introducing the ‘T2-FLAIR mismatch’ sign as a highly specific morphological feature of the IDH-mutant, 1p/19q non-codeleted molecular subtype of astrocytomas; Park *et al*.^15^ have used the Visually AcceSAble Rembrandt Images (VASARI) library in lower grade gliomas and shown that features like larger proportion of enhancing tissue, multifocal/multicentric distribution, and poorly marginated non-enhancing tumour tissue were independent predictors of an IDH1 wild type tumour. Though suitable for application in routine clinical settings, the morphological features utilize subjective MRI interpretation by readers commonly of variable experience, rather than quantitative or semi-quantitative image analysis. In theory, this might limit the reproducibility of the results.

Commonly, the analysis of advanced MRI results involves histogram analysis, which is considered a first-order statistical method where global features such as mean and standard deviation can be extracted from the average of pixel intensities. Second-order statistical features are measured based on the relationship between two pixels, whereas the higher-order statistical features are based on the relationship between more than two pixels. Therefore, first-order statistical methods do not convey spatial information, whereas the second and higher order statistical methods maintain spatial information and may reflect better the lesion heterogeneity. Texture analysis (TA) is a higher-order analysis and constitutes a mathematical method, which has shown preliminary potential to gain detailed insight into tissue composition^16–18^. Ideally, machine learning could be used to assist the diagnostic by enhancing its accuracy. Specifically, a computer algorithm could be trained on a set of training samples, to discover the most distinctive set of features samples from a large range of potential imaging biomarkers, whilst progressively eliminating non-discriminating lesion patterns in a recursive feature elimination manner.

Obviously, the moderate diagnostic accuracy of the conventional DWI metrics (based on the assumption of the Gaussian distribution) dictates the need to apply more elaborated solutions, such as DKI, for predicting the IDH mutational status. Moreover, the use of structural imaging for this purpose is usually confined within the usual visual assessment and texture features with machine learning have not been widely utilized. There is also scarce evidence for the use of DKI assisted by texture analysis with machine learning for imaging-based IDH phenotyping^13,19^. The purpose of this study is to validate the previous reports on DKI for IDH mutation status prediction and investigate, whether the diagnostic performance of DKI, either as stand-alone modality or combined with conventional structural imaging, may be enhanced by means of computerized texture analysis assisted by machine learning.

## Results

The final study population included 37 patients (21 males and 16 females, mean age 63.2 years ± 7.6 [standard deviation], age range 27–76 years). The median Karnofsky performance score (KPS) was 80 (range:70–90). Table 1 shows the clinical as well as the integrated histopathological and molecular findings in our cohort according to the 2016 WHO CNS tumor classification. There was no statistical difference in the age, gender and KPS between the IDH-mutant and wild-type tumours, between the astrocytomas and oligodendrogliomas, and between grade 2 and 3 tumours.

**Table 1:** Subgroups in the patient cohort classified according to the 2016 Update of the WHO Classification of Brain Tumors, and mean kurtosis as well as skewness values of the respective tumor subgroups.

Molecular parameters	Histology	WHO grade	n (median age; M/F)	Median Karnofsky score	Mean diffusional kurtosis ± SD	Skewness diffusional kurtosis ± SD
IDH1/2 mutation	Astrocytoma	2	11 (46;7/4)	90	516.19 ± 97.20	1.03 ± 0.91
	Anaplastic astrocytoma	3	4 (49;1/2)	80	482.69 ± 107.76	0.82 ± 0.77
	Oligodendroglioma	2	6 (52;2/4)	80	504.60 ± 40.65	1.66 ± 2.95
	Anaplastic oligodendroglioma	3	5 (46;2/3)	80	586.15 ± 64.70	−0.11 ± 0.74
	*Pooled*		*26 (48;12/13)*	80	521.81 ± 85.62	0.93 ± 1.61
IDH1/2 wild type	Astrocytoma	2	2 (50;1/1)	90	648.58 ± 171.56	0.88 ± 0.34
	Anaplastic astrocytoma	3	9 (49;2/1)	80	634.97 ± 137.77	2.05 ± 3.17
	*Pooled*		*11(50;3/2)*	80	637.45 ± 134.75	1.84 ± 2.88

### First-order statistics

The first-order (descriptive) DKI statistics (incl. average MDK values and their skewness in the segmented tumors) for the different subgroups are also given in Table 1. One-way ANOVA demonstrated a non-significant difference for average MDK and skewness among the different histological subgroups (p = 0.06 and 0.56, respectively). After classifying the tumors according to their IDH mutation status, the average skewness did not demonstrate significant difference among the two groups (p = 0.22). Nonetheless, the pooled IDH tumors demonstrated lower average MDK (p = 0.003), median MDK (p = 0.01), 5th percentile MDK (p = 0.02) and 95th percentile MDK (p = 0.008).

## Support vector machine analysis

### DKI first-order statistics biomarkers

DKI first-order statistics biomarkers (mean, median, standard deviation, kurtosis, 5th and 95th percentile) were entered SVM analysis to test the diagnostic performance of prediction of IDH mutation using a LOOCV to avoid over-fitting. In each iteration of the LOOCV, biomarkers are selected on the training set only using one-way ANOVA test with p < 0.05; an SVM was then trained based on the selected biomarkers and tested on the remaining example, thus there is no overlap between training and testing. Four statistical biomarkers (mean, median, 5th and 95th percentile) were consistently selected across different iterations of LOOCV. This resulted in 81.1% accuracy (sensitivity = 0.96, specificity = 0.45, AUC = 0.59).

### DKI and FLAIR texture biomarkers for gliomas WHO grade differentiation and IDH mutation

**Figure 1:**
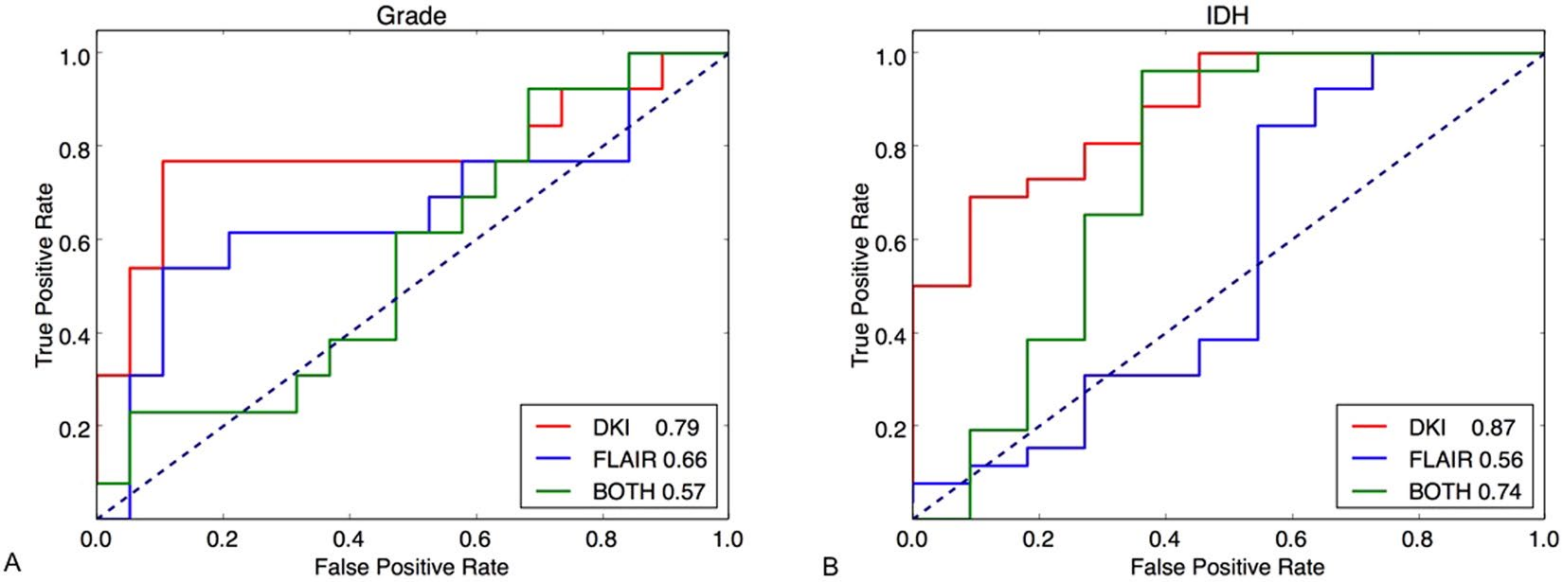
ROC curves and AUC values (in the legend) for the WHO gliomas grade (**A**) and the IDH mutation status (**B**). Three ROCs are shown in each case: using biomarkers from DKI (red), using biomarkers from FLAIR (blue) and using biomarkers from both DKI and FLAIR (green). The AUCs are also annotated in each experiment.

**Figure 2:**
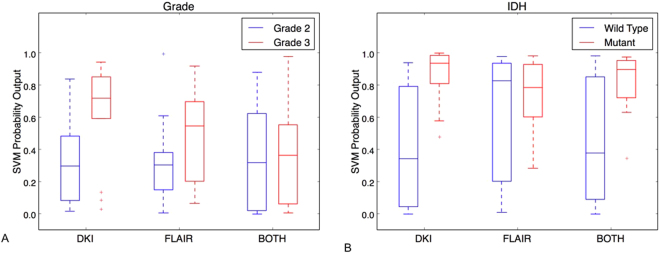
SVM probability outputs for WHO gliomas grade (**A**) and IDH mutation status (**B**) for the DKI and FLAIR as standalone modalities and for their combination. The predictive superiority of DKI as standalone modality is evident for both WHO tumor grade and IDH status tumor classification.

For the task of gliomas grade determination, gliomas WHO grade 2 was treated as “negative” and grade 3 as “positive”. The classification accuracy achieved by using 54 DKI biomarkers was 78.1% (sensitivity 0.77, specificity 0.79). The accuracy by using 54 FLAIR biomarkers was 68.8% (sensitivity 0.54, specificity 0.79). For the combined DKI and FLAIR 108 biomarkers, an accuracy of 50% was obtained (sensitivity 0.31, specificity 0.63). The ROC curves for three tests in this task are shown in Fig. 1A. The corresponding AUCs were 0.79 (DKI), 0.66 (FLAIR), 0.57 (both modalities), respectively. Using DKI biomarkers alone outperformed others. The AUCs of DKI and “both modalities” were significantly different (p = 0.01), while the other pairs (DKI vs FLAIR and FLAIR vs both) were not. The boxplots of SVM probability outputs for differentiating gliomas WHO grade using different modalities biomarkers are shown in Fig. 2A.

For IDH mutation determination task, wild-type status was treated as “negative” and mutant status as “positive”. The experiment using 54 DKI biomarkers resulted in a mutation prediction accuracy of 83.8% (sensitivity 0.96, specificity 0.55). The prediction accuracy by using 54 FLAIR biomarkers was 73% (sensitivity 0.88, specificity 0.36), and the classifier trained on both modalities (108) biomarkers achieved a prediction accuracy of 83.8% (sensitivity 0.96, specificity 0.55). Figure 1B shows the ROC curves for the classifiers trained in the three cases. The corresponding AUCs were 0.87 (DKI), 0.56 (FLAIR), 0.74 (both modalities) respectively, which indicates that using DKI alone outperforms the others. The AUCs of DKI and FLAIR were significantly different (p = 0.01), while the other pairs (DKI vs both and FLAIR vs both) were not. The boxplots of SVM probability outputs for determination of the IDH mutation status using different modalities biomarkers are shown in Fig. 2B.

### Biomarkers selection for gliomas WHO grade differentiation and IDH mutation

For a fair comparison, we applied RFE with RBF kernel for identifying the most four discriminative DKI texture biomarkers. As DKI is the phenotyping modality and shows better performance than FLAIR and combination of DKI and FLAIR based on the above experiments, the biomarker selection was then performed on 54 DKI biomarkers for both classification tasks of gliomas grade and IDH mutation status.

For gliomas grade determination, the selected 4 biomarkers are the 11^th^, 9^th^, 52^nd^ and 47^th^ biomarkers in the DKI biomarkers set, the according texture filter response for each selected biomarker for gliomas grade determination is shown in Table 2. For IDH mutation status determination, the selected biomarkers are the 11^th^, 14^th^, 42^nd^ and 7^th^ biomarkers in the DKI biomarkers set, the according texture filter response for each selected biomarker for IDH mutation status determination is shown in Table 3.

**Table 2:** The four most discriminative DKI-biomarkers selected by RFE for gliomas grading classification.

Index	Biomarker
11^th^	5th percentile value of Gaussian filter response
9^th^	standard deviation value of Gaussian filter response
52^nd^	kurtosis value of bar filter (scale = [12,4]) response
47^th^	5th percentile value of bar filter (scale = [6,2]) response

**Table 3:** The four most discriminative DKI-biomarkers selected by RFE for IDH mutation status.

Index	Biomarker
11^th^	5th percentile value of Gaussian filtered response
14^th^	median value of Laplacian of Gaussian filter response
42^nd^	95th percentile value of bar filter (scale = [3,1]) response
7^th^	mean value of Gaussian filter response

We then performed classification based on the selected 4 biomarkers shown in Table 2 for differentiating gliomas WHO grade and in Table 3 for determination of IDH mutation status, respectively, using the nested LOOCV. An accuracy of 75.0% (sensitivity = 0.69, specificity = 0.79, AUC = 0.85) was achieved for gliomas grade classification and an accuracy of 83.8% (sensitivity = 0.92, specificity = 0.64, AUC = 0.88) for IDH mutation status classification.

## Discussion

Preliminary evidence supports the potential role for DKI as a biomarker in the context of the new integrated glioma diagnosis^13,19^, but our study sought to test this hypothesis using texture features beyond the conventional statistical approaches. Compared to Hempel *et al*.^13^, we achieved remarkably better sensitivity (96% vs 82%) with a similar AUC (0.87 vs 0.85) but our specificity was considerably lower (0.55 vs 0.81). The different cohort composition might be the most important reason for this discrepancy. The IDH wild-type tumors in their study were predominantly GBMs (15 out of 16), whereas in our study the IDH wild-type tumours did not demonstrate GBM features, though some of them (most likely captured early during the natural history of tumour development) had EGFR amplification and TERT mutation, which are molecular hallmarks of GBMs. Histologically distinct GBMs have different intravoxel kurtosis features compared to the other astrocytomas and oligodendrogliomas, based on the different intrinsic heterogeneity^20,21^. Therefore, the high prevalence of IDH wild-type GBMs in the work of Hempel *et al*. is biased favoring high MK specificity in prediction of the IDH mutation status. On the contrary, our comparable in size IDH wild-type subgroup (n = 11) consisted of astrocytomas with indeterminate for grading imaging findings. This tumor class poses greater difficulties than conventionally radiologically diagnosed GBMs difficulties for IDH mutation stratification and is challenging the current imaging modalities. Thus, we firmly believe that texture feature analysis and SVM are valuable add-ons in the analysis and helped to meet the exigent demand for non-invasive molecular phenotyping of the IDH status.

We tested the diagnostic performance of the biomarkers from DKI, FLAIR and their combination of both for each task. It was noted that the difference between the AUCs using pairwise comparison of all ROC curves did not show significant difference between the DKI and FLAIR for gliomas grade determination and this might be due to the small number of patients, worth further exploration. However, in our experiments, the AUC of DKI (0.79) is higher than FLAIR (0.66) by a large margin (20% improvement). It is reasonable to conclude that using DKI biomarkers alone outperformed others. The same holds for IDH mutation determination with AUC of DKI (0.87) and both (0.74), which has 18% improvement. After all, the results using the 4 respective selected DKI biomarkers were very competitive in terms of accuracy compared to those using all 54 DKI biomarkers (gliomas grading: 75% vs 78.1%, IDH mutation status: 83.8% vs 83.8%) and higher AUC value (gliomas grading: 0.85 vs 0.79, IDH mutation status: 0.88 vs 0.87), which reflects the effectiveness of the feature selection procedure and also substantially simplifies the application, thereby maximizing its potential for clinical use.

Our DKI texture features are extracted from filters designed at multiple scales, orientations and frequencies, which was inspired by the study of the receptive fields in the primary visual cortex^22^. Distinct from the commonly used univariate statistical features, our filters capture the spatial information of a voxel in its local neighborhood in DKI images, where our bar and edge filter could detect the enhanced vascular structure of tumor typing at different scales, and capture its local context cues. Our features also differ from another set of texture features using popular Grey-Level Concurrence Matrix (GLCM) and its associated statistics, which usually considered features at a single scale (e.g., on raw intensities) and sensitive to image resolutions^23^. Our study has also demonstrated the superiority of using texture features for determination of IDH mutation status, given the same number of biomarkers (e.g., we selected 4 biomarkers in our study), the AUC value of using texture biomarkers has improved by 49% (0.88 vs 0.59) compared with that of using first-order statistical biomarkers.

The classification was performed using a SVM model with a nonlinear Gaussian kernel on all of the features. This approach leveraged the powerful machine learning model due to its capability of producing nonlinear separation hyperplane in the primal feature space, thus avoiding the assumption that distributions of data are linearly separable^24^. Our feature selection was carried out in a recursive elimination manner to find the best combination of DKI features for tumor grade or mutation prediction. By doing so, our method could avoid redundancy between selected features (the redundancy refers to the case where the two features essentially carry the same information) but include features which has complementary information for tumor grade or mutation prediction. This can be treated as an advantage compared with the methods that rank features individually^23,25^.

Our method used a rigor setting of nested LOOCV to avoid an optimistic performance estimate (for example, features are selected based on whole set or using only one loop, which usually results in an optimistic estimate of accuracy), and overfitting (i.e., selected features perform perfectly on training set, yet poorly on test set). The resulting feature sets will normally have robust generalisation performance on unseen images. SVM feature selection (e.g., recursive feature elimination-SVM) critically depends on having clean data since the feature outliers may play an essential role^25^. Also, the heterogeneity of patient data results in some inconsistency of selected features in each fold of LOOCV when the dataset is relatively small. This variation was alleviated by using the maximum voting across different folds in the inner LOOCV loop to reduce the effect of data noise.

In parallel with our work, an independent study by Eichinger *et al*.^26^, also used the texture feature based machine learning method to predict IDH genotype in newly diagnosed grade 2–3 gliomas with 95% accuracy in the validation cohort. However, our method differs significantly from theirs in three aspects: 1) different modalities; their study is based on b0 and fractional anisotropy diffusion (FA) images, while we explored DKI and anatomical FLAIR; compared to FA, DKI is a novel imaging technique with great potential; 2) different texture features; Eichinger *et al*. used local binary pattern (LBP) features, while we calculated multi-scale maximum response filter (MR8) features which is rotation invariant; 3) Eichinger *et al*. trained a neural network with a single hidden layer to predict IDH and used the Garson’s algorithm for feature selection, while we performed a recursive feature elimination based on support vector machine (RFE-SVM) for feature selection and IDH prediction.

Since this study was performed prospectively, it includes a comparatively small number of patients, the results presented here require confirmation in a larger study. The used DKI algorithm is restricted to an isotropic diffusion model, which may have introduced inaccuracy into the kurtosis tensor estimation. However, considering the anisotropic features on the kurtosis model would add to the post-processing time, which might be inexpedient for the dissemination of the method, whereas any diagnostic advance is doubtful^6^. To optimize DKI precision, our study adopted the technique proposed by Poot *et al*., which minimizes the mathematical lower bound (Cramer-Rao lower bound) on the variance of any unbiased estimator; the diagnostic gain of this process can be invested either to increase DKI estimation accuracy or image resolution^27^.

In conclusion, our findings expand the current scarce but promising evidence on the diagnostic ability of DKI to predict IDH mutational status, and by employing texture analysis and SVM on the DKI maps we rendered a small selection of biomarkers to distinguish satisfactorily IDH-mutant from IDH-wild-type tumors as well as grade II from grade III gliomas. Although the focus of this study is on gliomas, our approach demonstrated herein is expected to have applications in other tumor disease entities and encourage the larger-scale implementation in clinical studies, allowing for investigation of its accuracy.

## Methods

### Patient cohort

Between January 2015 and March 2017, 94 consecutive patients with suspected primary gliomas, who underwent multi-modality MRI (incl. DKI) as part of the pre-surgical workup, were prospectively examined. We aimed at morphologically (conventional MRI findings) presumably low-grade or indeterminate grade gliomas excluding tumors with intense contrast-enhancement, considerable perifocal edema and necrosis, which are universally encountered suggestive of glioblastomas. General exclusion criteria were also any contraindications to MRI exams, and agitated or non-cooperating patients.

Tumour diagnosis was based on histological examinations of surgical specimens, aided by immunohistochemical testing for known biomarkers (ATRX, IDH1 R132H) and molecular tests for rarer IDH 1 and all IDH mutations, 1p/19q co-deletion and TERT promoter mutations (see details in the *Histopathology* section). All histological and molecular studies were completed within 2 weeks of diagnosis and before initiating treatment. None of the patients received steroid treatments at the time of analysis.

The study was approved by the institutional review board (“St.Savvas” General Anti-Cancer and Oncological Hospital) and was conducted based on the principles of the International Conference on Harmonisation of Good Clinical Practice guidelines and according to the revised version of the Declaration of Helsinki. All patients provided written informed consent for the imaging surveys and the subsequent use of images for scientific and research purposes.

### Image acquisition

All exams were performed at a 3 Tesla (3 T) MRI scanner (Skyra, Siemens Healthineers, Erlangen, Germany). The conventional MR examination protocol included high-resolution 3D FLAIR and T1-weighted sequences before and after gadolinium administration (Gadovist®; Bayer-Schering, Germany). DKI was acquired using a spin-echo echo-planar imaging DWI sequence with the following parameters: *b*-values included 0, 500, 1000, 1500, 2000, and 2800 sec/mm^2^ with 30 directions and for each *b*-value diffusion encoding; TR 5900 ms; TE 95 ms; field of view 25 × 25 cm^2^; matrix 128 × 128; bandwidth, 965 Hz/pixel; slice thickness, 2.5 mm; number of signal averages, 2; and parallel imaging with a sensitivity encoding (SENSE) factor of 2. The total time of the DKI acquisition was 8 min 28 s.

### Image post-processing and analysis

The selection of the acquisition parameters for the kurtosis imaging was based on the suggestions of a recent consensus paper^28^. The mean DK (MDK) images were generated using in-house written software (R2014a MATLAB, MathWorks, Natick, MA, USA) and previously described models^28^. Parametric maps of MDK were calculated as the average of all 30 directions of the 6 b-values. The applied methodology, is based on the previous work by Jensen and Helpern^3^ and is briefly explained in the Supplement.

Image registration between the FLAIR-weighted images and MDK parametric maps was performed using the optimised automatic 3D registration tool in MIPAV (http://mipav.cit.nih.gov). Volumes of interest (VOIs) were manually drawn around the whole FLAIR abnormality. The tumor segmentations were performed in consensus by 2 board-certified neuroradiologists (each with 10-year experience in neurooncology imaging) who were blinded to the clinical diagnoses, using ITK-SNAP (http://www.itksnap.org)^29^. The tumor VOIs were then transferred MDK maps for texture analysis.

### Histopathology

All gliomas were diagnosed according to the WHO 2016 guidelines^30,31^. Initial stratification and diagnosis were achieved by morphological assessment and immunostaining for Ki67, ATRX, and IDH1 R132H^9^. Subsequently, all IDH R132H immuno-negative tumors underwent further characterization by Sanger sequencing of the established tumor-associated mutations of IDH1, IDH2, histone H3F3A, TERT promoter and BRAF genes^32^. A qPCR based copy number assay was used to determine 1p/19q status, EGFR amplification and 10q loss.

### Texture Feature Extraction

**Figure 3:**
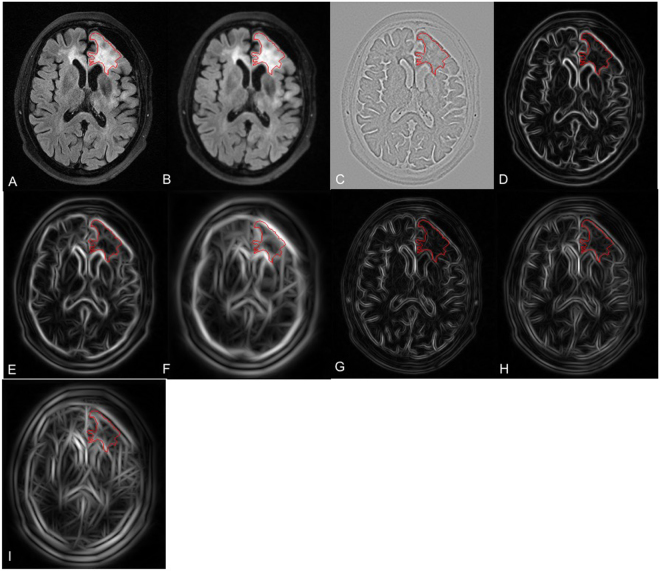
Visualization of MR8 filter responses on the mean kurtosis images in a patient with a glioma on the left frontal lobe. (**A**) original DKI image; (**B**) Gaussian response; (**C**) LoG response; (**D**–**F**) responses of edge filters at 3 scales; (**G**–**I**) responses of bar filters at 3 scales. The red curves are the tumor VOIs annotated by two experienced neuroradiologists in consensus.

**Figure 4:**
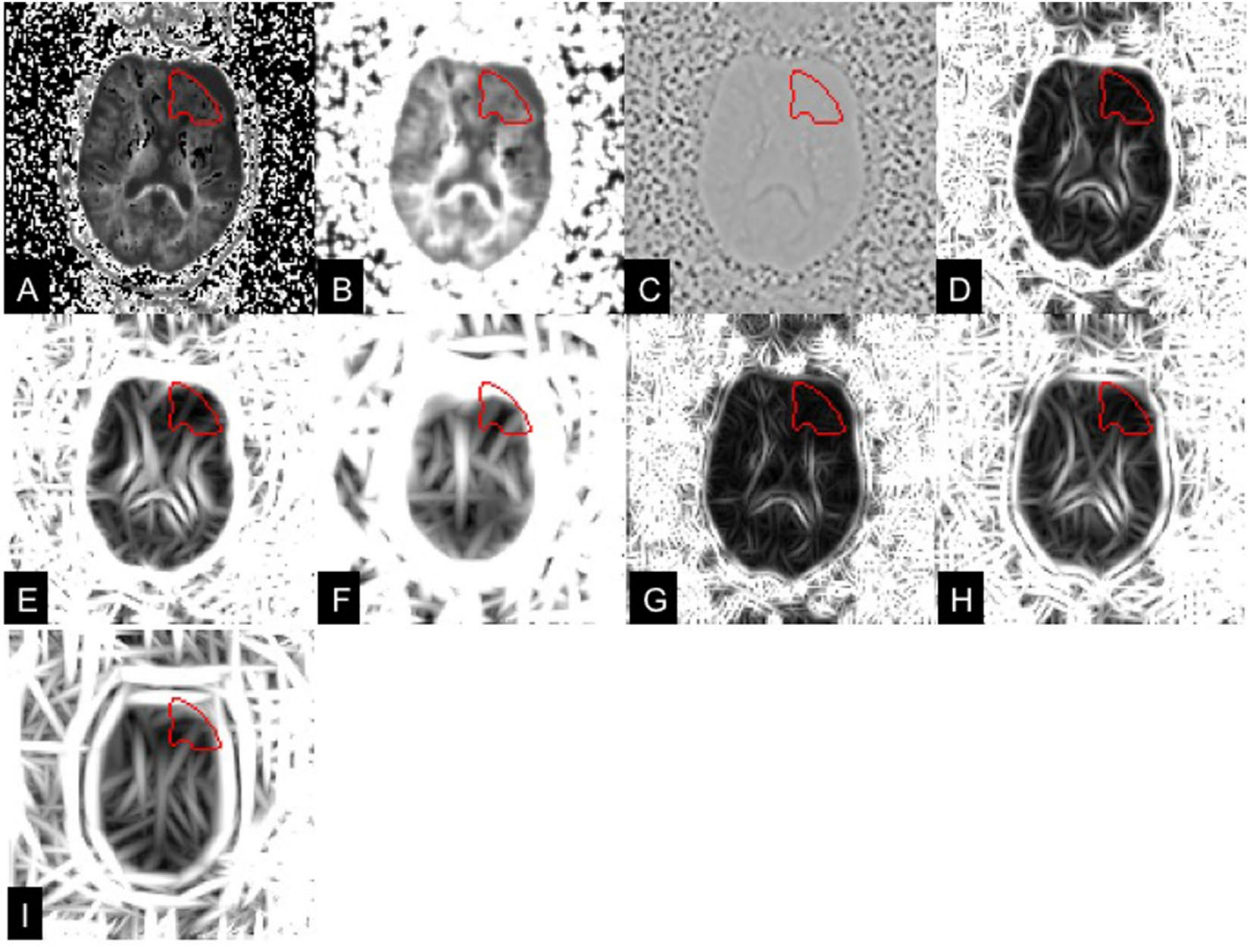
Visualization of MR8 filter responses on the FLAIR images in a patient with a glioma on the left frontal lobe. (**A**) original FLAIR image; (**B**) Gaussian response; (**C**) LoG response; (**D**–**F**) responses of edge filters at 3 scales; (**G**–**I**) responses of bar filters at 3 scales. The red curves are the tumor VOIs annotated by two experienced neuroradiologists in consensus.

The following features were extracted from both DKI and FLAIR VOIs: i) original intensity values; ii) responses of maximum response filter (MR8)^22^. Briefly, MR8 consists of 38 filters (one Gaussian, one Laplacian of Gaussian, 18 edge filters and 18 bar filters, which are at 3 scales and 6 orientations) and measures the maximum responses across 6 orientations for edge and bar filters, resulting in 8 rotation invariant filter responses. The orientations were set to [0°, 30°, 60°, 90°, 120°, 150°]. The standard deviation $\sigma$ was set to 1 for rotationally symmetric filters (Gaussian and Laplacian of Gaussian filter). The standard deviations of [${\sigma }_{x}$, ${\sigma }_{y}$] were set to [3,1], [6,2], [12,4] for anisotropic filters (edge and bar filters) at 3 scales. The responses of each filter for FLAIR and DKI images are illustratively shown in Figs 3 and 4. For each type of texture feature (e.g., intensity and each response of MR8), we calculated the *mean*, *median*, *standard deviation*, *kurtosis* and *5th* and *95th percentiles* as biomarker candidates, resulting in 54 biomarker candidates in each modality (FLAIR and DKI).

### Support vector machine analysis

Support vector machine (SVM) is a state-of-the-art classifier that constructs a hyperplane (in the primal or mapped space) as a decision boundary that, in our case, was used to separate the two groups (i.e. IDH-wild type and IDH-mutant tumors)^24^. A mathematical overview of the applied SVM algorithm is given in the Supplement. We conducted experiments for two binary classification tasks: 1) differentiating gliomas grade (2 vs 3); and 2) differentiating IDH mutation status (wildtype vs mutated). For each task, three tests were carried out: 1) 54 DKI biomarkers; 2) 54 FLAIR biomarkers; and 3) 108 biomarkers from both DKI and FLAIR. Support vector machine (SVM) with RBF kernel was used as the classifier. Different weights were assigned to different classes to handle the problem of class imbalance in both tasks (see the appendix for details). The SVM outputs (i.e., the distance to the hyperplane) were then calibrated into posterior probabilities using Platt scaling by training an additional sigmoid function^33^.

Due to limited number of patients, a nested leave-one-out cross validation (LOOCV) setting was used with the inner loop for parameter selection and the outer for model assessment, as the nested cross-validation is an almost unbiased estimate of the true error^34^. Specifically, the optimal parameters of SVM (C, γ) was found by grid search using the inner LOOCV and the outer one was used to assess the prediction performance. In this study, all the experiments including feature extraction, feature selection, classification were performed using MATLAB R2014a (MathWorks, Natick, MA).

### Biomarker selection by recursive feature elimination

We performed biomarker selection using recursive feature elimination based on SVM (RFE-SVM) to select the most discriminative biomarkers for each task^25^. The ranking criterion of each biomarker in RFE-SVM is based on its weight in SVM model, which indicates the importance of its contribution to the hyperplane. Recursive feature elimination iteratively eliminates the least important biomarker and retrain the SVM until reaching a pre-defined number of biomarker. Class weights were applied to address the class imbalance during each elimination procedure.

For each loop of nested LOOCV, we first selected N initial candidate biomarkers using RFE with RBF kernel. In order to reduce the influence of data outliers and noise, N ≥ 4 was set (e.g., N = 4, 5, 6 in our experiment setting). The chosen N initial candidate biomarkers from each loop were then put in a candidate pool. All the candidate biomarkers in the candidate pool were ranked according to the frequency of being selected during nested LOOCV loops with different N values. The top four ranked biomarkers in the candidate pool were subsequently treated as the *final* selected biomarkers.

Based on the selected biomarkers, two classification tasks were then performed using SVM with RBF kernel to evaluate the effectiveness of the selected biomarkers.

### Statistical Analysis

The Shapiro-Wilk test for normal distributions was applied for all continuous variables. The Kruskal-Wallis test, a nonparametric method for testing the equality of population medians among groups, was used on the selected biomarkers to determine whether the median feature value differed significantly between two groups. Chi-Square test of independence was used to detect any significant relationship between categorical variables, whereas parametric and non-parametric tests as well one-way ANOVA was utilised to test the hypothesis whether the average values differ among groups. Receiver operating characteristic (ROC) curves were generated for the DKI- and FLAIR-derived variables to determine sensitivity, specificity and area under ROC curve (AUC). The statistical significance of the difference between the AUCs was tested using pairwise comparison of all ROC curves^35^. Statistical significance was defined at p < 0.05. All analyses were performed with using MATLAB R2014a (MathWorks, Natick, MA).

## Electronic supplementary material

Supplement

## References

[1] Patterson DM, Padhani AR, Collins DJ (2008). Technology insight: water diffusion MRI–a potential new biomarker of response to cancer therapy. Nat Clin Pract Oncol.

[2] LaViolette PS (2014). Precise *ex vivo* histological validation of heightened cellularity and diffusion-restricted necrosis in regions of dark apparent diffusion coefficient in 7 cases of high-grade glioma. Neuro Oncol.

[3] Jensen JH, Helpern JA (2010). MRI quantification of non-Gaussian water diffusion by kurtosis analysis. NMR Biomed.

[4] Raab P, Hattingen E, Franz K, Zanella FE, Lanfermann H (2010). Cerebral gliomas: diffusional kurtosis imaging analysis of microstructural differences. Radiology.

[5] Van Cauter S (2014). Integrating diffusion kurtosis imaging, dynamic susceptibility-weighted contrast-enhanced MRI, and short echo time chemical shift imaging for grading gliomas. Neuro Oncol.

[6] Van Cauter S (2012). Gliomas: diffusion kurtosis MR imaging in grading. Radiology.

[7] Jiang R (2015). Diffusion kurtosis imaging can efficiently assess the glioma grade and cellular proliferation. Oncotarget.

[8] Tietze A (2015). Mean Diffusional Kurtosis in Patients with Glioma: Initial Results with a Fast Imaging Method in a Clinical Setting. AJNR Am J Neuroradiol.

[9] Reuss DE (2015). ATRX and IDH1-R132H immunohistochemistry with subsequent copy number analysis and IDH sequencing as a basis for an “integrated” diagnostic approach for adult astrocytoma, oligodendroglioma and glioblastoma. Acta Neuropathol.

[10] Elkhaled A (2012). Magnetic resonance of 2-hydroxyglutarate in IDH1-mutated low-grade gliomas. Sci Transl Med.

[11] Xiong J (2016). Combination of diffusion tensor imaging and conventional MRI correlates with isocitrate dehydrogenase 1/2 mutations but not 1p/19q genotyping in oligodendroglial tumours. Eur Radiol.

[12] Fudaba H (2014). Comparison of multiple parameters obtained on 3T pulsed arterial spin-labeling, diffusion tensor imaging, and MRS and the Ki-67 labeling index in evaluating glioma grading. AJNR Am J Neuroradiol.

[13] Hempel JM (2017). *In vivo* molecular profiling of human glioma using diffusion kurtosis imaging. J Neurooncol.

[14] Patel SH (2017). T2-FLAIR Mismatch, an Imaging Biomarker for IDH and 1p/19q Status in Lower-grade Gliomas: A TCGA/TCIA Project. Clin Cancer Res.

[15] Park, Y. W. *et al*. Prediction of IDH1-Mutation and 1p/19q-Codeletion Status Using Preoperative MR Imaging Phenotypes in Lower Grade Gliomas. *AJNR Am J Neuroradiol* (2017). 10.3174/ajnr.A5421 PMC7410710 29122763

[16] Brynolfsson P (2014). ADC texture–an imaging biomarker for high-grade glioma?. Med Phys.

[17] Chaddad A, Tanougast C (2016). Extracted magnetic resonance texture features discriminate between phenotypes and are associated with overall survival in glioblastoma multiforme patients. Med Biol Eng Comput.

[18] Itakura H (2015). Magnetic resonance image features identify glioblastoma phenotypic subtypes with distinct molecular pathway activities. Sci Transl Med.

[19] Hempel JM (2017). Histogram analysis of diffusion kurtosis imaging estimates for *in vivo* assessment of 2016 WHO glioma grades: A cross-sectional observational study. Eur J Radiol.

[20] Parsons DW (2008). An integrated genomic analysis of human glioblastoma multiforme. Science.

[21] Sui Y (2016). Differentiation of Low- and High-Grade Gliomas Using High b-Value Diffusion Imaging with a Non-Gaussian Diffusion Model. AJNR Am J Neuroradiol.

[22] Varma M, Zisserman A (2005). A statistical approach to texture classification from single images. Int J Comput Vision.

[23] Lee J (2016). Texture Feature Ratios from Relative CBV Maps of Perfusion MRI Are Associated with Patient Survival in Glioblastoma. AJNR Am J Neuroradiol.

[24] Burges CJC (1998). A tutorial on Support Vector Machines for pattern recognition. Data Min Knowl Disc.

[25] Guyon I, Weston J, Barnhill S, Vapnik V (2002). Gene selection for cancer classification using support vector machines. Mach Learn.

[26] Eichinger P (2017). Diffusion tensor image features predict IDH genotype in newly diagnosed WHO grade II/III gliomas. Sci Rep.

[27] Poot DH, den Dekker AJ, Achten E, Verhoye M, Sijbers J (2010). Optimal experimental design for diffusion kurtosis imaging. IEEE Trans Med Imaging.

[28] Rosenkrantz AB (2015). Body diffusion kurtosis imaging: Basic principles, applications, and considerations for clinical practice. J Magn Reson Imaging.

[29] Yushkevich PA (2006). User-guided 3D active contour segmentation of anatomical structures: significantly improved efficiency and reliability. Neuroimage.

[30] Louis DN (2014). International Society Of Neuropathology–Haarlem consensus guidelines for nervous system tumor classification and grading. Brain Pathol.

[31] Louis DN (2016). The2016 World Health Organization Classification of Tumors of the Central Nervous System: a summary. Acta Neuropathol.

[32] Brandner S, von Deimling A (2015). Diagnostic, prognostic and predictive relevance of molecular markers in gliomas. Neuropathol Appl Neurobiol.

[33] Platt, J. Probabilistic outputs for support vector machines and comparisons to regularized likelihood methods. In Smola, A.J. *Advances in large margin classifiers*, (MIT Press, Cambridge, Mass., 2000).

[34] Ambroise C, McLachlan GJ (2002). Selection bias in gene extraction on the basis of microarray gene-expression data. Proc Natl Acad Sci USA.

[35] Hanley JA, McNeil BJ (1983). A method of comparing the areas under receiver operating characteristic curves derived from the same cases. Radiology.

