# Supplementary material for: Texture analysis- and support vector machine-assisted diffusional kurtosis imaging may allow in vivo gliomas grading and IDH-mutation status prediction: a preliminary study
Source: Sci Rep. 2018 Apr 17;8:6108. doi: 10.1038/s41598-018-24438-4 (PMC5904150; doi:10.1038/s41598-018-24438-4)
Supplement: Supplementary file 1 — Supplement [file 41598_2018_24438_MOESM1_ESM.doc]

**Texture analysis- and support vector machine-assisted diffusional kurtosis imaging may allow in vivo gliomas grading and IDH-mutation status prediction: a preliminary study**

Sotirios Bisdas*^¶1-3^, Haocheng Shen^¶4^, Steffi Thust^¶1,2^, Vasileios Katsaros^1,5,6^, George Stranjalis^6^, Christos Boskos^6,7^, Sebastian Brandner^8,9^, Jianguo Zhang^4^

^1^ Department of Neuroradiology, The National Hospital for Neurology and Neurosurgery, University College London NHS Foundation Trust, London/UK

^2^ Department of Brain Repair and Rehabilitation, Institute of Neurology, University College London, London/UK

^3^ Institute of Healthcare Engineering, University College London

^4^ Computing, School of Science and Engineering, University of Dundee, Dundee/UK

^5^Department of Neuroradiology, General Anti-Cancer and Oncological Hospital of Athens “St.Savvas”, Athens/Greece

^6^Department of Neurosurgery, University of Athens, Evangelismos Hospital, Athens/Greece

^7^Department of Radiation Oncology, Athens Medical Center, Athens/Greece

^8^ Division of Neuropathology, The National Hospital for Neurology and Neurosurgery, University College London NHS Foundation Trust, London/UK

^9^ Department of Neurodegenerative Disease, Institute of Neurology, University College London, London/UK

**Supplement**

**Methods – Diffusion kurtosis imaging**

The DWI signal intensity, *S*, can be regarded as a function of the b-value, which for a Stejskal-Tanner sequence is defined by


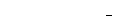
 **(1)**

where *g* is the proton gyromagnetic ratio, gamma is the amplitude of the diffusion sensitizing magnetic field gradient pulses, delta is the duration of the gradient pulses, and *D* is the time interval between the centres of the gradient pulses. According to the Taylor series ^1,2^


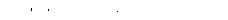
 **(2)**

where *D_app_* is the ‘apparent’ diffusion coefficient and *S_0_* (three lines) *S(0)*. Notably, it is assumed that both *D* and delta are fixed so that *b* is varied by changing *g*. In the short pulse duration limit delta 🡪 0, *D_app_* approaches the true diffusion coefficient *D* for a diffusion time *t =*
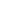
. Generally, if we assume the dependence on delta is small, we have the approximation


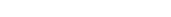
 **(3)**

The DKI model is based on the eqn (2) but includes explicitly the *O(b^2^)* term. The eqn (2) is expressed as


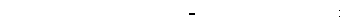
 **(4)**

corresponding to a cumulant expansion for the diffusion MR signal, where *Kapp* is the apparent diffusional kurtosis (unitless, equals 0 in the setting of completely Gaussian diffusion), which approaches the true kurtosis *K* in the limit of short pulse durations and contains specific information on the non-Gaussian diffusion behaviour ^3-5^. The parameter *D_app_* is the diffusion coefficient that is corrected to account for the observed non-Gaussian behaviour. The DKI extension of eqn (3) is


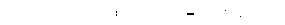
 **(5)**

where b-values are regarded sufficiently small so that the *O(b^3^)* term of eqn (4) is negligible. With this approximation, one can estimate both *D* and *K* by fitting to diffusion-weighted signal intensity data with three or more b-values in any gradient direction. The DKI model is parameterized by the diffusion tensor (DT) and kurtosis tensor (KT) from which several rotationally invariant scalar measures (e.g mean, axial, and radial diffusivity as well as fractional anisotropy (FA); and axial, radial, and mean kurtoses) ^6-8^. The interpretability of these metrics is influenced by the estimation accuracy of the tensors. Thus, any hampering factor (incl. noise, motion, and artifacts) can introduce errors that may propagate to render physically and/or biologically implausible tensor estimates ^9^. In this work, directionally-averaged *K_app_ (*later referred to as *MK)* were calculated using were estimated using unconstrained nonlinear least squares, which has been previously reported and demonstrated tissue-specific geometry for different brain regions ^7^.

**Methods – Support Vector Machine (SVM)**

In general, an SVM is a binary pattern recognition technique. The aim of SVM is to construct a hyperplane (e.g., decision boundary) that best separates the two groups by maximising a margin between the groups. Prediction of an unseen sample is performed by identifying which side of the hyperplane the data lies.

Theoretically, given a training set consisting N subjects (xi, yi), i=1,…,N; where xi constitutes a feature vector of each subject (e.g., the extracted biomarkers from DKI images), and yi represents a subject’s group label (e.g., -1 or 1), SVM finds the optimal hyperplane wTx + b = 0 by solving the following optimization problem:


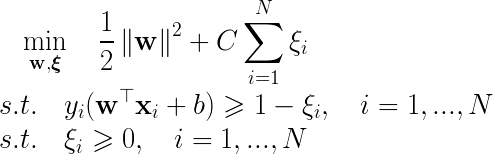


(1)

where C is a parameter controlling the trade-off between model complexity and training errors; ξi (i=1,…,N) are slack variables, which penalises each misclassified subjects as a function of distance from the hyperplane.

Formula (1) can be solved by optimizing the following dual form:


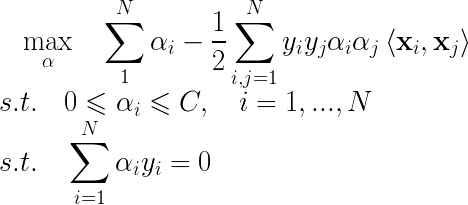


(2)

where αi is the Lagrange dual variable. A full derivation of the mathematics involved in SVMs can be found in [1]. For non-linear separable data, SVM uses a kernel <xi, xj> to map the data into a higher dimensional space where the data can be linearly separated by a hyperplane. One common choice for the kernel is the radial basis function (RBF):


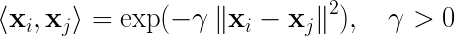


(3)

where γ is a free parameter controlling the width of the Gaussian kernel.

Once the SVM has identified the optimal hyperplane from the training data, the unseen test data x can then be classified based the sign of the decision function:


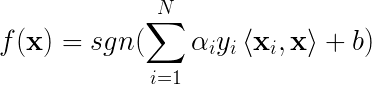


(4)

*Methods – Handling class imbalance*

The training phase of SVM is sensitive to class imbalance, an issue which occurs when one group has more subjects than the other. In this case the major group will overwhelm the correct training of the classifier, making the label of major group more likely to be predicted during testing. In our case, both tasks have imbalance data (e.g., 23 WHO grade 2 vs. 14 WHO grade 3 and 26 mutants vs. 11 wild-type). We addressed this problem by replacing the free parameter C with Cp and Cn for positive and negative classes respectively. Formula (1) then becomes:


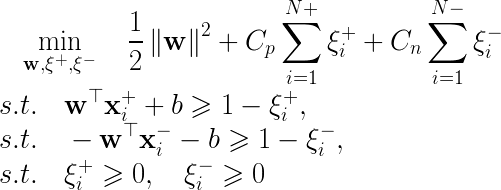
(5)

where xi+, xi- are positive and negative training examples in the training set; N+ and N- are the numbers of such examples; ξi+, i=1,…,N+ and ξi- are slack variables; Cp and Cn are set as Cp = (N+ + N-)/(2N+) × C0 and as Cn = (N+ + N-)/(2N-) × C0 . C0 was found by grid search.

**References**

**1.** Tanner JE. Transient Diffusion in a System Partitioned by Permeable Barriers - Application to Nmr Measurements with a Pulsed Field Gradient. *J Chem Phys.* 1978; 69(4):1748-1754.

**2.** Powles JG, Mallett MJD, Rickayzen G, Evans WAB. Exact Analytic Solutions for Diffusion Impeded by an Infinite Array of Partially Permeable Barriers. *P Roy Soc Lond a Mat.* 1992; 436(1897):391-403.

**3.** Tanner JE. Transient Diffusion in a Medium with Permeable Barriers. *Biophys J.* 1978; 21(3):A215-A215.

**4.** Liu C, Bammer R, Acar B, Moseley ME. Characterizing non-Gaussian diffusion by using generalized diffusion tensors. *Magn Reson Med.* 2004; 51(5):924-937.

**5.** Frohlich AF, Ostergaard L, Kiselev VG. Effect of impermeable boundaries on diffusion-attenuated MR signal. *J Magn Reson.* 2006; 179(2):223-233.

**6.** Basser PJ. Inferring microstructural features and the physiological state of tissues from diffusion-weighted images. *NMR Biomed.* 1995; 8(7-8):333-344.

**7.** Lu H, Jensen JH, Ramani A, Helpern JA. Three-dimensional characterization of non-gaussian water diffusion in humans using diffusion kurtosis imaging. *NMR Biomed.* 2006; 19(2):236-247.

**8.** Hui ES, Cheung MM, Qi L, Wu EX. Towards better MR characterization of neural tissues using directional diffusion kurtosis analysis. *Neuroimage.* 2008; 42(1):122-134.

**9.** Tabesh A, Jensen JH, Ardekani BA, Helpern JA. Estimation of tensors and tensor-derived measures in diffusional kurtosis imaging. *Magn Reson Med.* 2011; 65(3):823-836.
